# Anti-Nociceptive and Anti-Inflammation Effect Mechanisms of Mutants of Syb-prII, a Recombinant Neurotoxic Polypeptide

**DOI:** 10.3390/toxins11120699

**Published:** 2019-12-01

**Authors:** Chunli Li, Mengqi Ban, Fei Bai, Jianzhao Chen, Xiaoquan Jin, Yongbo Song

**Affiliations:** Department of Pharmacology, Shenyang Pharmaceutical University, Shenyang 110016, China; 104040205@syphu.edu.cn (C.L.); banmonkey1@126.com (M.B.); 15653250971@163.com (F.B.); chenjianzhao@live.cn (J.C.); jxq918@126.com (X.J.)

**Keywords:** Syb-prII mutants, analgesia, anti-inflammation, sodium channel, MAPKs

## Abstract

Syb-prII, a recombinant neurotoxic polypeptide, has analgesic effects with medicinal value. Previous experiments indicated that Syb-prII displayed strong analgesic activities. Therefore, a series of in vivo and vitro experiments were designed to investigate the analgesic and anti-inflammatory properties and possible mechanisms of Syb-prII. The results showed that administered Syb-prII-1 and Syb-prII-2 (0.5, 1, 2.0 mg/kg, i.v.) to mice significantly reduced the time of licking, biting, or flicking of paws in two phases in formalin-induced inflammatory nociception. Syb-prII-1 inhibited xylene-induced auricular swelling in a dose-dependent manner. The inhibitory effect of 2.0 mg/kg Syb-prII-1 on the ear swelling model was comparable to that of 200 mg/kg aspirin. In addition, the ELISA and Western blot analysis suggested that Syb-prII-1 and Syb-prII-2 may exert an analgesic effect by inhibiting the expression of Nav1.8 and the phosphorylation of ERK, JNK, and P38. Syb-prII-1 markedly suppressed the expression of IL-1β, IL-6, and TNF-α of mice in formalin-induced inflammatory nociception. We used the patch-clamp technique and investigated the effect of Syb-prII-1 on TTX-resistant sodium channel currents in acutely isolated rat DRG neurons. The results showed that Syb-prII-1 can significantly down regulate TTX-resistant sodium channel currents. In conclusion, Syb-prII mutants may alleviate inflammatory pain by significantly inhibiting the expression of Nav1.8, mediated by the phosphorylation of MAPKs and significant inhibition of TTX-resistant sodium channel currents.

## 1. Introduction

The International Association for the Study of Pain (IASP) defines pain as an unpleasant sensation and emotional experience associated with substantial or potential tissue damage [1]. Pain is classified in two types: nociceptive pain and pathological pain [2].

Nociceptive pain is caused by intense irritation to evoke the sensation of pain and comes after direct tissue damage. However, pathological pain, which may have somatic symptoms such as in sensory hypersensitivity and persistent pain, is divided into inflammatory pain and neuropathic pain. Neuropathic pain is defined as pain directly caused by damage or disease of the somatosensory system, including peripheral fibers (Aα, Aδ, and C fibers) and central neurons [3,4]. Pathological pain is quite a problem mainly because under this condition hypersensitivity results in maladaptive problems and can be prolonged after recovery from the cumulative damage [5].

For another reason, pathological pain has always been of concern as a clinical challenge due to a lack of effective solutions. Well-known analgesics, such as opioids and non-steroid anti-inflammatory drugs (NSAIDs), involve some side effects which cannot be ignored [6]. In addition, NSAIDs are reported to have adverse effects in terms of inhibiting osteoblast growth [7]. In this case, traditional Chinese medicines (TCMs) have become new strategies, drawing the attention of a number of researchers. As is known, TCMs have good therapeutic effects and few side effects in many cases due to their multi-component, multi-factorial, and multi-target characteristics. Furthermore, the components of those natural medicines are also highly bioactive and have potential in a variety of new drugs.

In China, *Buthus martensi* kirsch (Bmk), has been used to treat epilepsy, pain, and tumors, etc., as part of TCM. Its active ingredient comes from the venom of the Bmk’s tail. The venom of Bmk consists of a highly complex mixture of many substances such as peptides, enzymes, free amino acids, lipids, amines, heterocyclic components, inorganic salts, and other unknown ingredients. To date, over 1500 species of scorpion species have been discovered, divided into 18 families [8]. BmK is most widely distributed in China and has been utilized in TCM for thousands of years. Based on some studies it was found that toxic scorpion peptides have substantial bioactivity in ion channels, especially sodium channels [9]. Previous electrophysiological studies in sodium channels confirm that TTX-resistant sodium channels are closely related to the molecular mechanism of nociception [10]. Besides, some cytokines can regulate the expression of sodium channels via mitogen-activated protein kinases (MAPKs) downstream [11].

**Figure 1:**
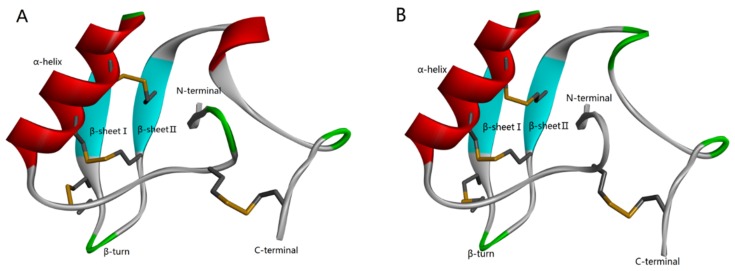
Homology modeling of Syb-prII-1 (**A**) and Syb-prII-2 (**B**).

According to the homology analysis of sequence and structure (Figure 1), Syb-prII protein, which includes 62 amino acid residues, forms part of a β-anti-excitatory neurotoxin isolated from the venom of Bmk. In general, β-scorpion toxins are composed of 60–65 amino acid residues, which form conservative structure with α-helix and three antiparallel β-strands by four pairs of cross-linked disulfide bonds. From the perspective of sequence and structure conservation, these two peptides should belong to the β-anti-excitatory neurotoxin, which mainly targets sodium ion channels. By binding to receptor site 4, β-scorpion toxins shift the voltage dependence of activation of VGSCs to cause subthreshold opening of channels and reduce the peak current amplitude. Our previous work demonstrated strong analgesic effects of Syb-prII-1 and -2 in an acetic acid-induced writhing test. Therefore, with an aim of exploring the mechanism of the potent anti-inflammatory and analgesic effects, we performed work and discussed it comprehensively.

## 2. Results

### 2.1. Purification of the Syb-prII Protein

**Figure 2:**
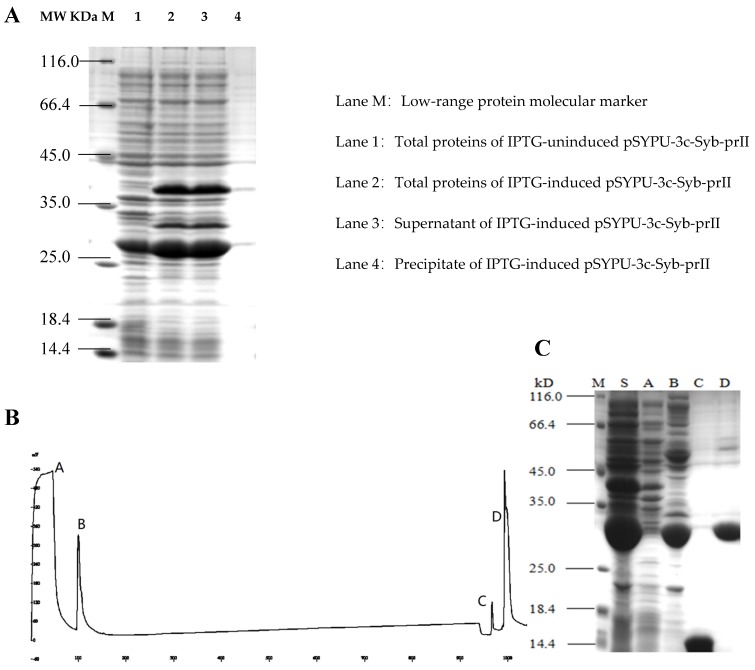
Purification of recombinant Syb-prII by Chelating Sepharose Fast Flow. (**A**) 12.5% SDS-PAGE analysis of recombinant Syb-prII expressed in *E. coli*; (**B**) Metal-chelating affinity chromatography; (**C**) 15% SDS-PAGE analysis. Lanes A–D represent the corresponding peaks of buffer A–D in (**B**); Lane M: protein molecular weight marker (116.0, 66.2, 45, 35, 25, 18.4, and 14.4 kDa); Lane S: Supernatant of IPTG-induced pSYPU-3c- Syb-prII.

To improve the solubility of the Syb-prII, the intein protein and TrxA were constructed in the expression vector pSYPU-3C. The induction of intein self-cleavage was conducted by a pH shift, and the optimum condition was pH 5.5 at 20 °C for 12 h in this study. SDS–PAGE analysis indicated that the eluates containing target protein finally eluted with buffer C (Figure 2B, elution fraction C peak), and produced a single band (Figure 2C, Line C). The large fragment (His6-Intein, about 27 kD) of the fusion protein (His6-Intein-Syb-prII, about 37 kD) cleaved was observed mostly in fraction D (Figure 2B). Compared with lane S and B in Figure 2, the result showed that the cleavage efficiency was approximately 90%.

### 2.2. Effects of Syb-prII-1 and -2 in Inflammatory Animal Models

**Figure 3:**
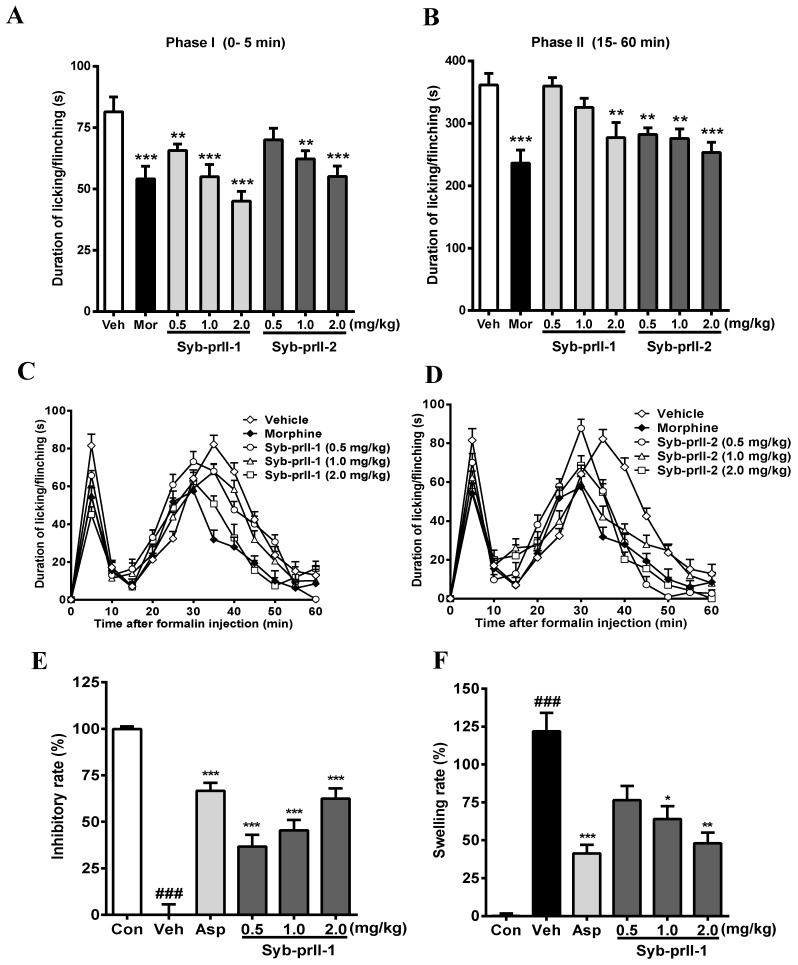
Evaluation the antinociceptive effect of Syb-prII-1 and Syb-prII-2 in phase I (**A**) and phase II (**B**) of the formalin test. (**C**,**D**) show the time course of Syb-prII-1 and Syb-prII-2 in formalin-induced inflammatory nociception. The swelling rate (**E**) and inhibitory rate (**F**) of Syb-prII-1 in xylene induced ear swelling in mice. ### *p* < 0.001 versus control group, * *p* < 0.05, ** *p* < 0.01, *** *p* < 0.001 versus vehicle group. Data are shown as mean ± SEM.

The results we obtained showed that the intra-plantar formalin injection produced a spontaneous pain response in the control group, accompanied by licking, flinching, and shaking behaviors as well as local inflammatory reaction, dyspraxia, and emotional responses. These nociceptive behaviors and emotional responses were alleviated after the administration of morphine, Syb-prII-1, and Syb-prII-2. The inhibitory effects of Syb-prII-1 and Syb-prII-2 in phase I and phase II pain of the formalin model are shown in Figure 3. In the inflammatory phase, the dose of phase I Syb-prII-1 was 2.0 mg/kg, which was stronger than morphine, and the inhibition of phase II Syb-prII-2 was better than that of Syb-prII-1 (Figure 3A,B). Syb-prII-1 showed inhibitory effects of 19.4% (0.5 mg/kg), 32.5% (1.0 mg/kg), and 44.8% (2.0 mg/kg) in phase I and 0.5%, 10.1%, and 23.4% in phase II, respectively. Syb-prII-2 showed inhibitory effects of 14.0% (0.5 mg/kg), 23.6% (1.0 mg/kg), and 32.4% (2.0 mg/kg) in phase I and 22.0%, 23.7%, and 30.0% in phase II, respectively.

The analgesic effect of the drug on formalin-induced pain over time is shown in Figure 3C,D. As seen in Figure 3E,F, the results showed that Syb-prII-1 could produce inhibitory effects in a dose-dependent manner and at the dosage of 2.0 mg/kg Syb-prII-1 could did as well as 200 mg/kg aspirin. This indicated that Syb-prII-1 could inhibit the ear edema by reducing the level of inflammatory responses during the acute inflammation.

### 2.3. Syb-prII-1 Lowers the Secretion Levels of Inflammatory Factors

**Figure 4:**
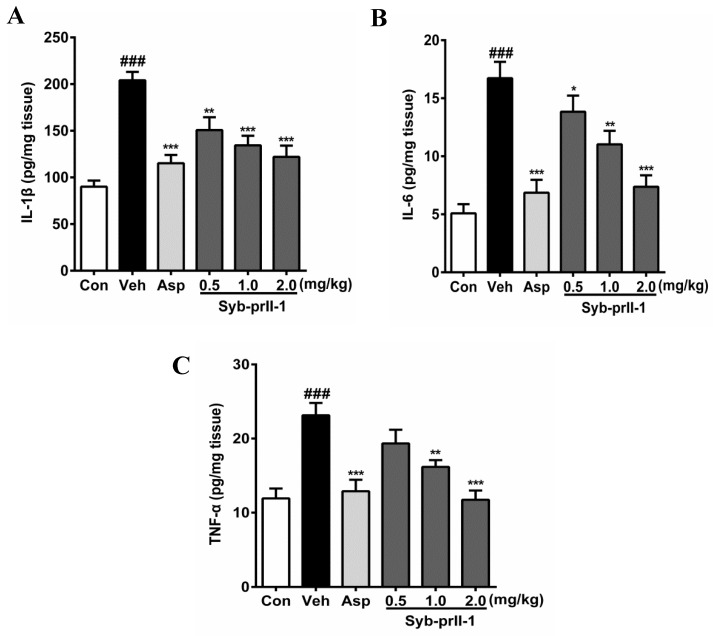
After administration of aspirin (200 mg/kg) or Syb-prII-1 (0.5–2.0 mg/kg), the inflammatory cytokines IL-1β (**A**), IL-6 (**B**), and TNF-α (**C**) in the tissue of the ear swelling model were measured by an ELISA kit. Each bar represents the mean ± SEM. The graphic symbols denote the significance levels when compared with control groups. ### *p* < 0.001 compared with control group, ** *p* < 0.01, *** *p* < 0.001 compared with vehicle group. Data were shown as mean ± SEM.

The expression levels of inflammatory factors in tissues can be seen in Figure 4. In comparison with the blank control, the levels of IL-1β, IL-6, and TNF-α in the vehicle were promoted significantly; in addition, the expression level of IL-6 reached 3.28 times of that in the blank control. Moreover, as compared to the vehicle, both aspirin and Syb-prII-1 inhibited increases in inflammatory factors in xylene-induced ear edema in a dose-dependent manner.

### 2.4. Syb-prII-1 Reduces the Expression of the TTX-R Sodium Channel, Nav1.8, and Nav1.9

**Figure 5:**
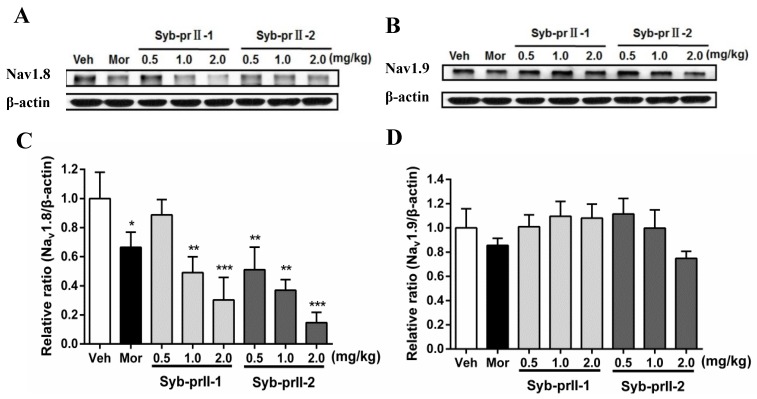
Changes of Nav1.8 and Nav1.9 protein expression in L4–6 DRGs among groups. The bands are representative for the expression of Nav1.8 (**A**) and Nav1.9 (**B**) in DRGs after injection of formalin. The quantitative data were for the expression of Nav1.8 (**C**) and Nav1.9 (**D**). The fold change for the density of Nav1.8 and Nav1.9 was normalized to β-actin for each sample (*n* = 3). Data are shown as mean ± SEM. * *p* < 0.05, ** *p* < 0.01, *** *p* < 0.001 compared with vehicle group.

As seen in Figure 5, in comparison with vehicle, the expressions of Nav1.8 in L4-L6 DRGs in the morphine and Syb-prII-1/2 groups all decreased in a dose-dependent manner. Meanwhile, the levels of expressions of Nav1.9 at Syb-prII-1/2 groups rose slightly, indicating that Syb-prII-1/2 could not have the same impact on the expressions of Nav1.9 as they had in Nav1.8.

### 2.5. Syb-prII-1 Reduces the Phosphorylation of MAPKs

**Figure 6:**
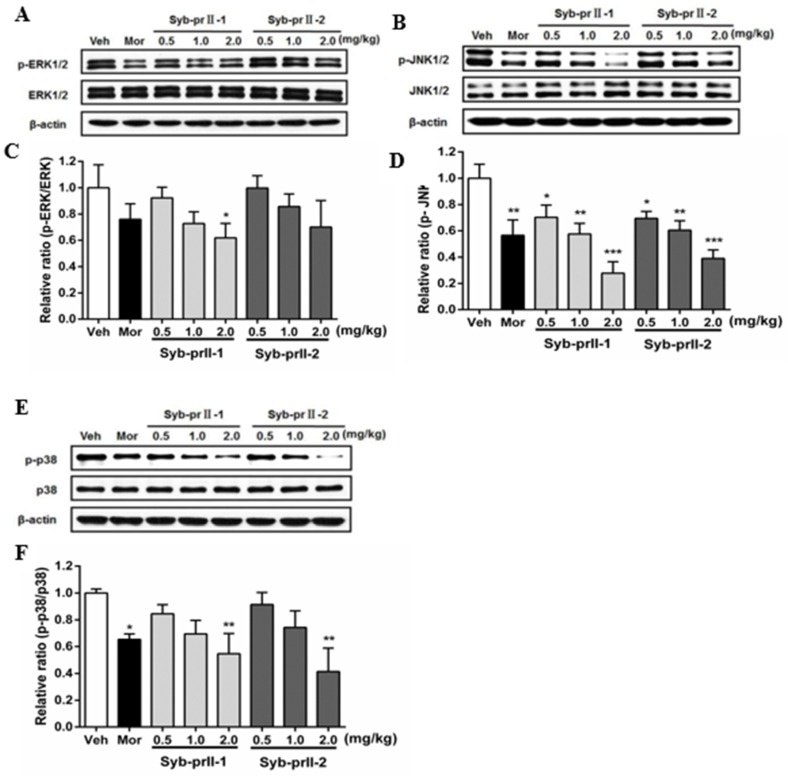
Changes the expressions and phosphorylations of ERK/JnK/P38 map kinases in L4–6 DRGs among groups (**A**,**B**,**E**). The exact fold changes were exhibited as a relative ratio of the phosphorylations with the total expressions (**C**,**D**,**F**). Data are shown as mean ± SEM. * *p* <0.05, ** *p* < 0.01 compared with vehicle group. For each band *n* = 3.

As known, MAPK pathways play an important role in both nociceptive pain and pathological pain, thus we explored the mechanism of the underlying therapeutic effect of Syb-prII-1/2. The results showed that Syb-prII-1 could inhibit the expression of P-ERK in a dose manner, especially at the dosage of 2.0 mg/kg, while Syb-prII-2 could not provide an ideal inhibition on the expression of P-ERK relevant to the standard level (Figure 6A,C). The expressions of P-JNK and P-P38 were impressionable and were remarkably decreased by morphine, Syb-prII-1, and Syb-prII-2. In addition, Syb-prII-1 and -2 produced significant effects in a dose-dependent manner at any dosage on p-JNK (Figure 6B,D), but only at the dosage of 2.0 mg/kg on P-P38 (Figure 6E,F).

### 2.6. Effects of Syb-prII-1 on Nav1.8 Currents

**Figure 7:**
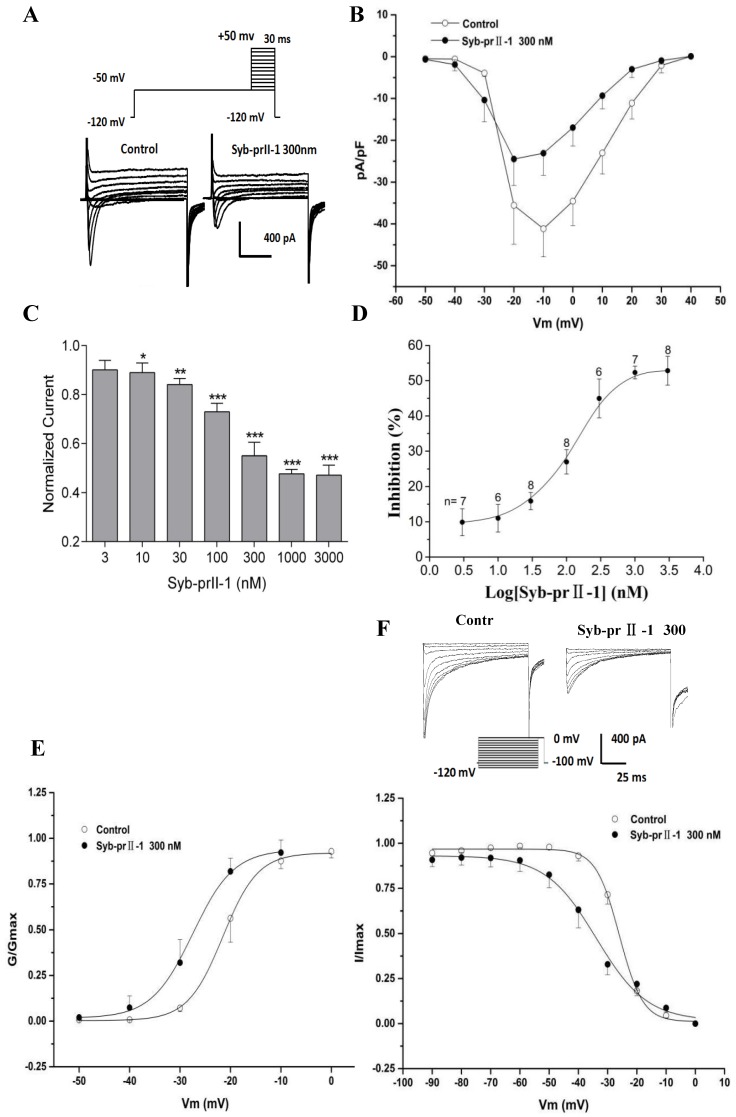
Effects of Syb-prII-1 on the Nav1.8 channel in small-diameter DRG neurons. (**A**) Representative traces of Nav1.8 activation currents in the Syb-prII-1 (300 nM) and control groups. (**B**) The effect of 300-nM Syb-prII-1 on the I–V curves of Nav1.8 currents. (**C**,**D**) Syb-prII-1 inhibited the Nav1.8 current in a concentration-dependent manner with an IC_50_ of 133.42 nM. (**E**) The effect of 300 nM Syb-prII-1 on the Nav1.8 steady-state activation curve. (**F**) Representative inactivation currents traces and steady-state inactivation curve of Nav1.8 in the absence or presence of 300 nM Syb-prII-1. *** *p* < 0.001 compared with vehicle group. Data are shown as mean ± SEM.

The extracellular application of 4-AP, TEA, La^3+^, and TTX, as well as the addition of Cs^+^ to the recording electrode, blocked voltage-dependent calcium, potassium, and TTX-s currents. Figure 6 shows the normalized peak amplitude of I_Nav1.8_ induced by 100 ms depolarizing test pulses from −50 mV to +50 mV in 10-mV increments after 500 ms prepulses at −50 mV in the same DRG neurons from a holding potential −100 mV, and in this case I_Nav1.9_ are not activated because Nav1.9-mediated currents are activated at hyperpolarized potentials (Figure 7A) [12]. After pre-administration of Syb-prII-1, in comparison with the control, Syb-prII-1 offered obvious concentration-dependent inhibitory effects. It was observed that 10 nM peptide could significantly decrease the Nav1.8 current amplitude by 11.0 ± 3.9% (*p* < 0.05), and 1000 nM of Syb-prII-1 could reach the peak inhibitory effect of a reduction of 52.3 ± 1.8% (*p* < 0.001). It was found that 3000 nM of Syb-prII-1 could not provide further inhibition, with a value of 52.8 ± 4.1% (*p* < 0.001); furthermore, at this dose Syb-prII-1 presented cytotoxic activities (Figure 7C,D).

### 2.7. Effects of Syb-prII-1 on Activation and Inactivation Kinetics of Nav1.8 Currents

Follow the previous protocol, a series of Nav1.8 currents were recorded. Setting the current densities as ordinates and the pulse voltages as abscissa, we obtained the current density–voltage (I–V) curves of Nav1.8 (Figure 7B). We observed that after treating 300 nM of Syb-prII-1, the peak amplitude of I_Nav1.8_ lowered, the activation potential of the peak current lowered from −10 mV to −20 mV, and the curves shifted towards depolarization. Meanwhile, we set the G/G_max_ as ordinates and the pulse voltages as abscissa, according to the Boltzman function: G/G_max_=1/{1+exp[(V_1/2 act_ − V)/k]}, where G_max_ stands for the maximum value for conductance, V_1/2_ for the potential at which activation is half-maximal, and k is the slope factor. We obtained the steady-state-activation fitting curve of Nav1.8. The dose of 300 nM Syb-prII-1 shifted the curve towards the hyperpolarization by +6 mV compared to pre-dose. This dosage also lowered the V_1/2act_ from −21.5 ± 0.2 mV to −27.5 ± 0.7 mV and the slope factor K increased from 3.5 ± 0.3 to 4.1 ± 0.5 (Figure 7E). This all revealed that treating 300 nM Syb-prII-1 could accelerate the activation of Nav1.8.

To obtain the inactivation currents of Nav1.8, after holding potential at −100 mV we offered a series of depolarization steps of pre-pulses which proceeded over 500 ms ranging from −120 mV to 0 mV in 10 mV increments, and testing pulses at 0 mV with a duration of 100 ms after which the potential was converted back to −100 mV. Thus, we obtained the inactivation currents of Nav1.8. According to the Boltzman function I/Imax = 1/{1+exp[(V−V_1/2inact_)/k]}, where I_max_ stands for the peak sodium current, V_m_ for the preconditioning pulse potential, V_1/2inact_ is the potential at which I is half-I_max_, and k is the slope factor, we obtained a Nav1.8 steady state inactivation curve. It could be observed that after treating 300 nM of Syb-prII-1 that this curve would significantly shift towards hyperpolarization by +8 mV. In addition, compared with pre-dose, the V_1/2inact_ decreased from −26.0 ± 0.3 mV to −33.9 ± 1.3 mV, and factor K changed from 3.4 ± 0.3 to 8.7 ± 1.2 (Figure 7F). This indicated that treating 300 nM of Syb-prII-1 could also accelerate the procedure of inactivation of the Nav1.8 sodium channels in the small-diameter DRG neurons.

### 2.8. Effects of Syb-prII-1 on the Frequency-Dependent Relationship of Na_v_1.8 Currents

**Figure 8:**
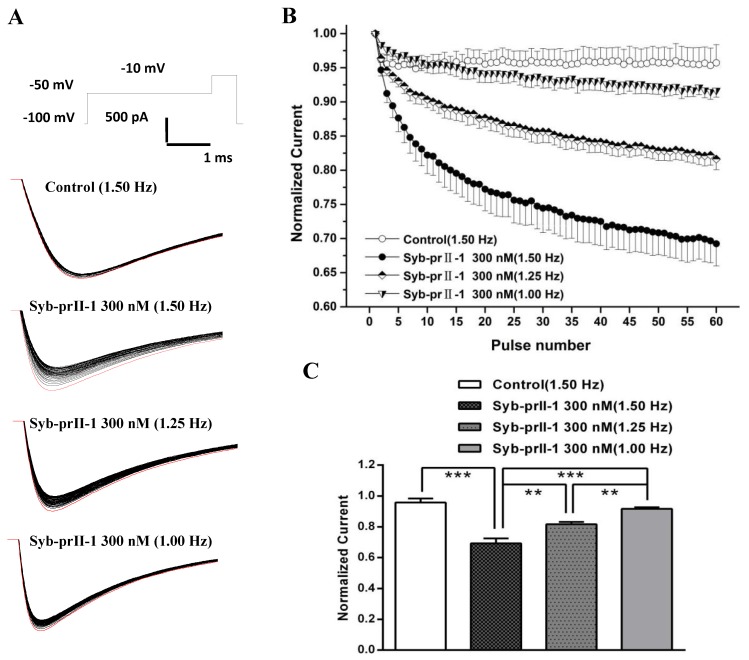
Effects of Syb-prII-1 on the frequency-dependently relationship of Nav1.8 currents in small diameter L4–6 DRG neurons. (**A**) The Nav1.8 current was evoked by 60 pulses at −10 mV. The first Nav1.8 current is red. (**B**) A 300-nM Syb-prII-1 frequency-dependently inhibits Nav1.8 currents. (**C**) The current at the 60th pulse normalized to the current of the respective first pulse. ** *p* < 0.01, *** *p* < 0.001. *n* = 6 per group.

Based on the properties of Nav1.8 channel proteins, we investigated whether Syb-prII-1 has a dependent blocking effect to further explore the mechanism of action. Stimulation was repeated 60 times with different stimulation frequencies to obtain a series of Nav1.8 currents (Figure 8A). Then, the stimulation frequency of 1.5 Hz was used to record Nav1.8 current as the blank control before administration. Syb-prII-1 was added at a final concentration of 300 nM. The Nav1.8 current was recorded by sequentially applying stimulation pulses of different stimulation frequencies of 1.0 Hz, 1.25 Hz, and 1.5 Hz. The magnitude of the current was normalized by the ratio between the Nav1.8 current amplitude of each impulse and the Nav1.8 current amplitude of the corresponding first impulse (Figure 8B,C). After adding Syb-prII-1 (300 nM), the Nav1.8 current was recorded using a stimulation frequency of 1.50 Hz. The Nav1.8 current recorded by the first stimulus was the largest, and the peak Nav1.8 current at the 60th stimulus decreased to 69.2 ± 3.2% of the first stimulus. Compared with the control group (95.7 ± 2.7%), there was a significant difference (*p* < 0.001), indicating that Syb-prII-1 was dependent on the Nav1.8 current of small-diameter DRG neurons. Under the same conditions, the larger the stimulation frequency, the more obvious the decrease in the peak value of Nav1.8 current. There was also a significant difference in the comparison between the different stimulation frequencies. This indicates that Syb-prII-1 has a frequency-dependent blockade of Nav1.8 current in small-diameter DRG neurons.

### 2.9. Effects of Syb-prII-1 on Nav1.9 Currents

**Figure 9:**
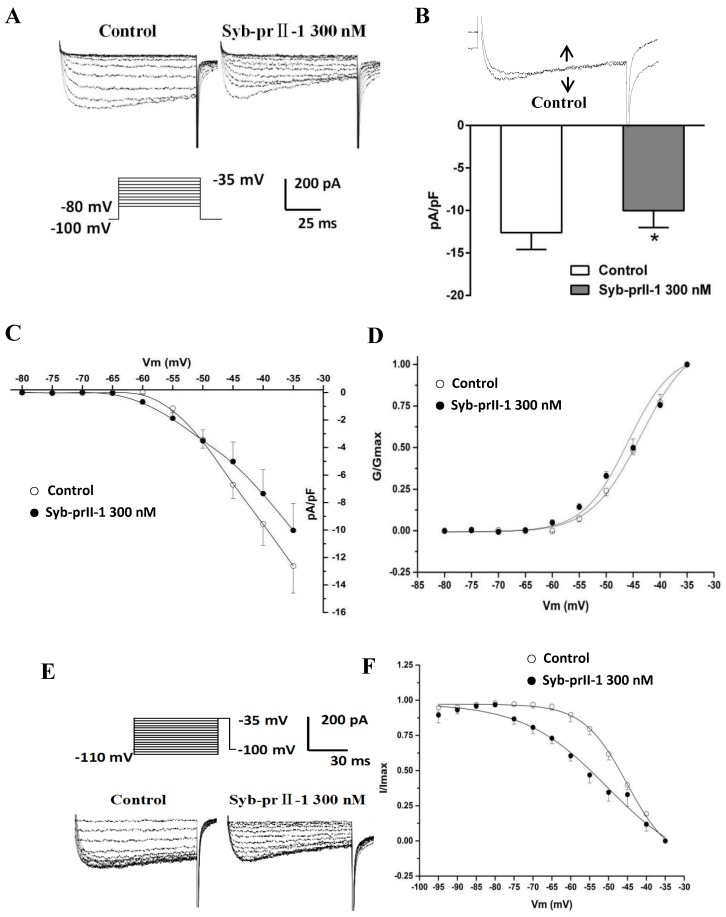
Effects of Syb-prII-1 on Nav1.9 in small diameter DRG neurons. (**A**) Histogram showing 300 nM Syb-prII-1 inhibited the peak amplitude of Nav1.9 currents at 22.5 ± 5.7%. (**B**) The effect of 300 nM Syb-prII-1 on the current density–voltage (I–V) curves of Nav1.9 currents. (**C**) Representative traces of Nav1.9 activation currents in the absence or presence of 300 nM Syb-prII-1. (**D**) The effect of 300 nM Syb-prII-1 on the Nav1.9 steady-state activation curve. (**E**) Representative traces of Nav1.9 inactivation currents in the absence or presence of 300 nM Syb-prII-1. (**F**) The effect of 300 nM Syb-prII-1 on the Nav1.9 steady-state inactivation curve. *** *p* < 0.001 compared with vehicle group. *n* = 6. Data are shown as mean ± SEM.

To examine the effects of Syb-prII-1 on I_Nav1.9_, we recorded the normalized peak amplitude of I_Nav1.9_ induced by 100 ms depolarizing test pulses from −80 mV to −35 mV in 10-mV increments. The results showed that 300 nM of Syb-prII-1 could remarkably inhibit the currents of Nav1.9 on the L4–6 DRG neurons by 22.5 ± 5.7%, *p* < 0.05 (Figure 9A,B). However, it is notable that compared with the effects on Nav1.8 (45.0 ± 5.5%, *p* < 0.001), at the same dosage of Syb-prII-1 the inhibition on Nav1.9 was far weaker. Set the current densities as ordinates and the pulse voltages as abscissa, we obtained the current density–voltage (I–V) curve of I_Nav1.9_ (Figure 9C). It could be observed that treating 300 nM of Syb-prII-1 advanced the activation of Nav1.9, but provided few impacts on the peak current activation potentials.

### 2.10. Effects of Syb-prII-1 on Activation and Inactivation Kinetics of Nav1.9 Currents

Again, steady-state activation curve of Nav1.9 was fitted according to the Boltzmann function G/G_max_ = 1/{1 + exp [(V_1/2 act_ − V)/k]}, where G_max_ stands for the maximum value for conductance, V_1/2_ for the potential at which activation is half-maximal, and K is the slope factor. On the same cell, treating 300 nM Syb-prII-1 shifted the curve towards the hyperpolarization direction and lowered V_1/2act_ from −43.6 ± 0.5 mV to −46.0 ± 1.5 mV; it also changed slope factor k from 4.7 ± 0.3 to 4.1 ± 0.7 (Figure 9D). All these revealed that Syb-prII-1 could accelerate the activation procedure of Nav1.9 which expressed on the small-diameter DRG neurons.

Similarly, after holding the potential at −100 mV, we offered a series of depolarization steps prepulses which proceeded for 700 ms, ranging from −110 mV to −35 mV in 5-mV increments and testing pulses at −35 mV with a duration of 100 ms, after which the potential converted back to −100 mV. We recorded the inactivation currents of Nav1.9. We set I/I_max_ as ordinates and the pulse voltages as abscissa, based on the Boltzman function I/I_max_ = 1/[1 + exp[(V−V_1/2inact_)/k]], where I_max_ stands for the peak sodium current, V_m_ for the preconditioning pulse potential, V_1/2inact_ is the potential at which I is half-I_max_, and k is the slope factor. We thus obtained the fitting curve of inactivation of Nav1.9. It is remarkable that compared with pre-dose, treating 300 nM of Syb-prII-1 shifted the inactivation curve of Nav1.9 by 5 mV (Figure 9E,F). In this case, the V_1/2inact_ changed from −45.1 ± 0.7 mV to −50.4 ± 4.1 mV, and the slope factor k changed from 5.8 ± 0.4 to 11.0 ± 2.8. This indicated that procedure of inactivating Nav1.9 was significantly accelerated by Syb-prII-1.

## 3. Discussion

### 3.1. The Anti-Inflammatory Effects of Syb-prII

In our test, administration of 2.5% formalin elicited a biphasic pain as described [13,14], in phase I (0~5 min) and phase II (15~60 min). The initial phase is mainly because formalin directly activated of nociceptive neurons by formalin [15]. The phase II is thought to be involved the inflammatory response to the injury and central neural system’s participation [16]. Generally speaking, centrally acting drugs inhibit both stages [17], while peripherally acting drugs only inhibit phase II [18]. As a result, we found that Syb-prII-1 was more effective in phase I than in phase II and Syb-prII-2 inhibited both phases the same, but weaker than Syb-prII-1 in phase I. It is worth noting that, in phase II, at the dosage of 0.5 mg/kg, Syb-prII-1 only provided an inhibition of 0.53%. However, when we raised the dosage to 1.0 mg/kg, the inhibition came up to 10.06%.

Inflammatory response is very complex, as this course involves many signaling molecules and immune cells [19]. Generally, when bodies receive noxious stimuli, acute inflammation is activated at an early stage. In this stage, vasodilatation occurs and vascular permeability rises and blood volume descends. At the same time, the local permeation of cytolymph and proteins will induce swelling [20]. Swelling is a very common pathological change and also a symptom of acute inflammation. Clinically, anti-inflammatory agents play a role in a strategy of inhibiting edema. Ear edema caused by xylene is one of the classic inflammatory models, and the inflammatory mechanism may involve inflammatory mediators to promote vasodilatation and increase vascular permeability [21]. Syb-prII-1 was very effective in inhibiting swelling in a dose-dependent manner. Therefore, our present data confirmed a significant anti-inflammatory effect of Syb-prII-1, which might reduce the local dimensions of inflammation mediators. In combination with data of formalin test, we could postulate Syb-prII-1 contributed to inflammatory analgesic effects mainly via anti-inflammation.

Such cytokines as IL-1β, IL-6, and TNF-α are essential in the procedure of inflammatory pain; IL-1β and TNF-α can excite the primary nociceptive neurons directly [22]. Also, these cytokines are known to be involved in many signal patterns, such that TNF-α can up-regulate the expression of Nav1.8 and Nav1.9 [23,24], and IL-10 can down-regulate voltage-gated sodium channels [25]. Our results of ELISA showed that Syb-prII-1 could decrease the expression of IL-1β, IL-6, and TNF-α. This indicated that Syb-prII-1 had an ability to prevent the generation of inflammation directly.

### 3.2. Syb-prII-1 Regulates Neuronal Excitability of DRG Sensory Neurons via Nav1.8 and Nav1.9

Our previous work revealed that Syb-prII-1 and Syb-prII-2 seem to be regulators of sodium channels. Based on the important role of Nav1.8 and Nav1.9 in the pain, Western blotting was chosen to test the effect of Syb-prII-1 and -2 on the populations of Nav1.8 and Nav1.9 in DRG neurons. Further, we used electrophysiology tests to testify the role of the alteration of the electrophysiology characters of Nav1.8 and Nav1.9.

Scorpion venom has been proved to be very fertile, with many active ingredients which could be practically potential regulators of pain. Syb-prII-1 has been proved to have effects on voltage-gated sodium channels. There are two subtypes of voltage-gated sodium channels resistant to tetrodotoxin (TTX), Nav1.8 and Nav1.9. It has been reported that Nav1.8 sodium channels were exclusively expressed in the subpopulation of primary sensory neurons, for example trigeminal and DRG neurons [26,27]. Studies have shown that changes in the expression, trafficking, and function of these two TTX-resistant sodium channels are the basis for neuronal hyperexcitability observed in the pain-like animal models [28]. Injection of carrageenan in the hind paws of mice can make the expression of mRNA and Nav1.8 increase in the DRG [29,30]. In a spinal nerve ligation (SNL) model, increased expression of Nav1.8 in the L4 nerve was observed [31]. Nav1.8 was increased after CFA injection [32]. In Nav1.8-blocked mice, allodynia induced by complete Freund’s adjuvant (CFA) could be reversed [33]. All these indicate that peripheral nerve injuries have an ability to alter the translation of Nav1.8, facilitate Nav1.8 to transfer from intact cell body to axons. This reveals Nav1.8 must be deeply involved in both peripheral neuropathic and inflammatory pain, but we cannot estimate it exactly. For this reason, these neurons which express Nav1.8 have been identified as a major factor in seizures and hypersensitivity under chronic disease conditions, thus intervening in Nav1.8 channel functionality and ablating the population of Nav1.8 by pharmacological and genetic tools have been rational strategies in variety of pain conditions. Our data supported that in this inflammation case, analgesic effects accompanied with a decreasing population of Nav1.8, as well as a lower activity and function of Nav1.8.

Rather than injecting carrageenan, CFA could raise the expressions of Nav1.9 and its mRNA significantly [34]. Knocking out the gene of Nav1.9 weakens the allodynia in many inflammation models, such as the formalin test, carrageenan model, CFA model, and PGE_2_-induced pain model [35]. However, after sciatic nerve axotomy, spinal nerve ligation (SNL), and spinal nerve injury (SNI), the expressions of Nav1.9 and its mRNA as well as its current density all decrease [36]. Intrathecal injection of ASODN of Nav1.9 cannot prevent or reverse the neuropathic pain induced by SNL [31]. Allodynia and thermal hyperalgesia still exist after knocking out the gene of Nav1.9 in SNL and SNI [37]. Thus, no inference about the ability of Nav1.9 to alter neuropathic pain can be confirmed, though obviously Nav1.9 is an important part of inflammatory pain. However, our present data support that Syb-prII-1 and -2 could not inhibit the expression of Nav1.9. This reveals the analgesic effects that Syb-prII-1 and -2 provided perhaps had little relevance with the inhibition of expression of Nav1.9.

TTX-resistant sodium channels are also deeply involved in the generation and propagation of action potentials, therefore regulating such channels affects neuronal excitability [38]. Nav1.8 in small-diameter DRG neurons has a high activation threshold and slow inactivation kinetics, contributing to the awakening of depolarizing currents [39], and Nav1.9 is involved in the forming process of resting potential [19]. Our work in electrophysiology showed that Syb-prII-1 could suppress Nav1.8 and Nav1.9 currents, and decrease the procedure of activation and inactivation kinetics of Nav1.8 and Nav1.9, thus testifying that Syb-prII-1 suppressed the function of such sodium channels to impede the peripheral nociceptive signal transmission. We infer that this might be the major role that Syb-prII-1 directly plays in analgesia effects.

### 3.3. The Regulation of Syb-prII on Nav1.8 and Nav1.9 via MAP Kinases

Some studies have shown that voltage-gated sodium channels can be regulated with some intracellular signal molecules through some signal pathways. It has been shown that P38 MAPK could be activated in DRG on inflammation or nerve injury [40,41]. There is also evidence that with the increase of expression of P38, current density of TTX-resistant and TTX-sensitive sodium channels both increased [42]. Activation of P38 pathways can only change the current density but not the gating properties [43]. However, ERK 1/2 pathways will elicit the activation and inactivation curves of Nav1.7 shifting towards hyperpolarization, increasing the frequency of action potentials, but do nothing to the current densities [11]. Thus, MAPKs tend to target specific sodium channels with totally different regulations.

It has been reported that MAPKs are very important to neuronal plasticity and regulation of inflammation. The latter phase of the biphasic pain caused by formalin is on the basis of the increasing population of the inflammation mediators. Later in this phase, ERK pathways in central nucleus were activated, which may be responsible to contralateral allodynia [44]. Then as a result, inhibiting this process will prevent the contralateral allodynia thoroughly as well as the homolateral inflammatory hyperalgesia. Allodynia induced by SNL can be prevented by intrathecal injection of the MEK inhibitor PD98059 [45]. In short, we know ERK pathways are essential in both inflammatory pain and neuropathic pain. Phosphorylation seemed to play an critical role for the up-regulation of Nav1.8 [46]. Similarly, analgesic levels can be suppressed by P38 inhibitors via intrathecal administration in neuropathic pain and inflammatory pain models [47]. Pathways of P38 are also activated because of pro-inflammatory cytokines and cell stress. Thus, P38 also plays a role in inflammatory responses. In addition, JNK pathways have been proved to be able to suppress hyperalgesia but not to reverse it. It seems that activation of JNK pathways is only involved in the early developments of allodynia [48]. Our results showed that Syb-prII suppressed both the phosphorylation and expressions of ERK1/2, JNK, and P38 MAP kinases. Based on these results, Syb-prII might also reduce pain in direct regulation in MAP kinases and have secondary impacts on TTX-resistant sodium channels.

## 4. Conclusions

In conclusion, we postulate that Syb-pII-1 has an analgesic effect by suppressing the expression and phosphorylation of MAPK pathways, reducing the population of inflammatory factors. Meanwhile, Syb-prII-1 inhibited the TTX-R sodium ion channels and exhibited much higher inhibitory effects on Nav1.8 compared with Nav1.9. This result suggested that Nav1.8 might be involved in the analgesic mechanism of Syb-pII-1, and may also contribute to its analgesia effects.

## 5. Materials and Methods

### 5.1. Animals

Female Sprague–Dawley rats (150–180 g) and Kunming mice (18–22 g) were purchased from the Animal Experimental Center of Shenyang Pharmaceutical University for the experiment. Animals were acclimated for several days in a standard laboratory environment and fed standard laboratory animal feed and water. All animal treatment programs comply with international ethical guidelines and the regulations of the Animal Ethics Committee of Shenyang Pharmaceutical University, China (SCXK (Liao) 2010-0001).

### 5.2. Strains

Plasmid pSYPU-3c, pNJU-ANEP, and *E. coli* strain BL21 (DE3) were stored in our laboratory and used for cloning and expression. Restriction endonucleases, Taq DNA polymerase, and T4 DNA ligase were purchased from TaKaRa (Dalian, China). The primers were synthesized by JINSITE (Nanjing, China). Chelating Sepharose Fast Flow was purchased from GE Healthcare (Pittsburgh, USA). The pSYPU-3c vector containing the Syb-prII gene was constructed in our laboratory.

### 5.3. Construction and Verification of the Expression Vectors pSYPU-3c/Syb-prII

The target gene encoding Syb-prII was amplified from using pNJU-ANEP as a template with the oligonucleotide primers Syb-prII-F and Syb-prII-R (Table 1) by PCR method, and then the PCR products were digested with EcoRI/SapQI and cloned into the pSYPU-3c separately to produce pSYPU-3c-Syb-prII-1 and pSYPU-3c-Syb-prII-2.

### 5.4. Transformation and Verification of the Vectors

pSYPU-3c-Syb-prII-1 and pSYPU-3c-Syb-prII-2 were transformed into *E. coli* strain BL21 (DE3). Transformants were cultured overnight at 37 °C with shaking in Luria–Bertani (LB) medium containing ampicillin (50 µg/mL). Positive clones were screened and identified by DNA sequencing using T7 promoter primers.

### 5.5. Expression of Recombinant Syb-prII

Positive transformants were inoculated in fresh LB broth containing ampicillin (50 μg/mL) at 37 °C and grown to mid-log phase (OD600 = 0.6–0.8). Then, IPTG was added to a final concentration of 0.2 mM and further grown for 4 h at 37 °C. Cells were pelleted at 3500 rpm for 20 min, washed two times, and resuspended in 40 mL 0.05 M phosphate-buffered saline (pH 8.0). The suspension was sonicated on ice. The lysate was then centrifuged at 12,000 rpm for 20 min at 4 °C. The supernatant and pellet were separated and analyzed by SDS-PAGE to test the solubility.

### 5.6. Purification of the Recombinant Scorpion Syb-prII

The supernatant, which was obtained after sonication, was loaded onto a Chelating Sepharose Fast Flow column (1.0 × 6.5 cm) equilibrated with buffer A (0.05 M PBS pH 8.0) at a flow rate of 0.2 mL/min. Then, the column was washed with buffer A at a 1 mL/min flow rate until A280 reached the baseline. Non-specific proteins and lipid were removed with two column volumes of buffer B successively; next, the column was washed with buffer B (pH 5.5) to perform intein splicing for 12 h at 20 °C. Then, the target protein was eluted with buffer C and buffer D (0.05 M EDTA) at a flow rate of 1.0 mL/min, and the elution peak was concentrated using Pall stirred ultra-filtration cells (3-kDa membrane) and identified via SDS–PAGE and collected.

### 5.7. Formalin Test

The formalin test was performed as described [49]. Each group of mice was administrated with normal saline, morphine (2.0 mg/kg), and Syb-prII-1/2 (0.5, 1.0, 2.0 mg/kg) via intravenous injections of the tail at a dosage of 0.1 mL/10 g, respectively, half an hour before receiving 2.5% formalin solution (20 μL) via subcutaneous injection in the ventral left hind paw. Right after injection, all the mice were placed in a transparent box (11 cm × 11 cm × 16 cm), and a 45° mirror was placed underneath to observe the paws of the mice. At the same time, an observation of mice for an hour was taken and the frequency of licking, flinching, and shaking of paws in a period of 5 min was recorded as indicative of nociception.

### 5.8. Xylene-Induced Ear Edema in Mice

According to the method described previously [17], aspirin (200 mg/kg) was given to the mice orally and normal saline and Syb-prII-1 (0.5, 1.0, 2.0 mg/kg) were given via intravenous injection to the tail. Thirty minutes later, to evoke inflammatory response, 20 μL xylene were administered to the right ear of each mouse, and the left ear was used as a control. One hour after administration, the mice were sacrificed through cervical dislocation. Two mouse ears of 7.0 mm diameter were taken for biopsy, perforated, and weighed. The degree of edema was assessed by the difference in weight between the tissues of the two ears of the same mouse. The inhibition rate of mouse ear swelling is calculated as follows (calculated as a percentage):

Inhibition (%) = (ear swelling control − ear swelling drug)/ear swelling control × 100%

All tissue were preserved at −80 °C after being weighed.

### 5.9. Biochemical Parameters

Tissue of xylene-induced ear swelling was added into a PBS buffer solution, sonicated, and centrifuged (1000× *g*, 20 min). The supernatant was collected according to ELISA kits to measure the contents of IL-1β, IL-6, and TNF-α protein. Briefly, 50 µL standard set from the sample (10 µL) + sample diluent (40 µL) groups were pipetted into the wells of a microtiter plate to measure proinflammatory cytokine levels and then incubated for 1 h at 37 °C. After washing five times, 50 µL of the chromogen solution A and 50 µL of the chromogen solution B were mixed and added, and incubated at 37 °C for 10 min in the dark. Then, 50 µL of the stop solution were added, and the color in the well turned from blue to yellow. Finally, the optical density at 450 nm was calculated by an automated Coulter microplate reader. The sample concentration is calculated from the OD value.

### 5.10. Extraction of Protein and Western Blot Analysis

The bilateral L4–L6 DRG from the mouse formalin test was dissected and total protein was extracted by homogenization in ice-cold lysate buffer and then centrifuged at 12,000 rpm for 15 min at 4 °C. Subsequently, the supernatant was collected and the protein level was quantitatively analyzed by the BCA method. The molecular weight of the protein is shown in Table 2.

Samples (target protein) were aspirated with a micro-syringe and loaded in each lane, and then underwent electrophoretic separation by 12% denaturing polyacrylamide gel electrophoresis (SDS-PAGE). The separated protein was transferred to a PVDF membrane. The membrane was transferred to a buffer containing 5% skim milk powder-TBST buffer and shaken at room temperature for about 1 h on a low-speed shaker. The membrane was removed from the blocking solution and washed with TBST. Subsequently, the membrane was incubated in the first antibody overnight at 4 °C. The membrane was removed the next day; after washing the TBST buffer sufficiently, a second antibody was added. After incubating for 1 h at room temperature, it was washed three times in TBST buffer. The target proteins were detected by enhanced chemiluminescence (ECL kit). Band analysis was performed using β-actin as an internal reference using Syngene gel analysis system software.

### 5.11. Preparation of DRG Neurons

DRG neurons were prepared as described above [50]. After deeply anesthetizing the SD rats, DRG neurons in the L4–L6 region of the lumbar spine were taken and placed in iced DMEM/F12 medium. Each sample of DRG tissue was cut into small pieces, placed in an ampoule containing 1 mL of digestive juice, digested for 15 min in a 37 °C incubator, and shaken once every 3 min. After the digestion was terminated, culture medium (0.3 mL) was added to the DRG tissue. Digested DRG tissue was triturated by a pipette (200 μL). After the tissue settled, the suspension containing DRG neurons was aspirated. This was planted on acid-washed coverslips which had been coated previously with 0.01% poly-L-lysine. This was followed by incubation in a 37 °C incubator until the cells were attached. Then a patch-clamp experiment was performed within 2–10 h.

### 5.12. Electrophysiology

Eager to clarify the specific alterations in TTX-resistant sodium channels in DRG neurons of Syb-prII, experiments of electrophysiology were set up and a patch clamp was chosen. Experiments were performed at room temperature. Those small-diameter DRG neurons with smooth and intact membranes were selected under the vision of a Nikon TE2000-U inverted microscope. Patch-clamp electrodes were prepared by a P-97 puller with a proper resistance of 3~5 MΩ and filled with pipette solution (in mM: CsCl 140, TEA-Cl 10, EGTA 10, HEPES 10, pH = 7.2, storage at −20 °C). Currents were recorded by an AxoPatch 200B amplifier (Axon Instruments, Foster City, CA, USA). All signals were filtered and data were stored digitally and analyzed by Clampex 10.0 and Clamfit 10.0 software. After the patch clamp was established, DRG neurons were transferred into extracellular fluid (in mM: NaCl 140, MgCl2 1, CaCl2 3, KCl 5, TEA-Cl 10, 4-AP 1, CdCl2 0.2, HEPES 10, glucose 10, pH = 7.3) and small-diameter DRG neurons were selected and held at designated potentials and given a step voltage stimulation according to relevant protocols. Subsequently, record current (TTX-resistant sodium channel). The current density was calculated from the peak current density and the battery capacitance (Cm) ratio. The conductance (G) was calculated according to the equation G = I/(Vm − Vr) (I: peak current amplitude, Vm: test potential, Vr: reverse potential of current).

### 5.13. Statistical Analysis

Data were measured and processed using Clampex 10.0 and Clampfit 10.0 software. Statistical analysis and mapping of experimental data were performed using SPSS 21.0, Origin 9.0, and Graph Pad Prism 6.02 software. The experimental results are expressed as Mean ± SEM. The paired-sample *t*-test was used for comparison between groups, and a significant difference was considered when *p* < 0.05.

## Figures and Tables

**Table 1:** Primers used to construct pSYPU-3c-Syb-prII ^a^.

Name	Oligonucleotide Sequence (5′-3′)	Orientation
Syb-prII-1-F	GGTGGTTGCTCTTCCAACGATGGATATATAAGAGGAAGTAACGGGATGCAAG	Sense
Syb-prII-2-F	GGTGGTTGCTCTTCCAACGATGGATATATAAGAGAGAAGAGATGGATGCAAG	Sense
Syb-prII-R	CCGGAATTCTTAGCCACCGCATGTATTACTTTCAG	Antisense

^a^ The underlined GAATTC and GCTCTTC sequences represent the EcoRI and SapQI restriction enzyme sites, respectively.

**Table 2:** Antibody and its strip size.

Antibody	Molecule Weight	Vendor
Nav1.8	220 kDa	Abcam
Nav1.9	202 kDa	Alomone
P-ERK1/2	42 kDa (up) 40 kDa (down)	Cell Signaling
ERK1/2	42 kDa (up) 40 kDa (down)	Cell Signaling
P-JNK1/2	54 kDa (up) 46 kDa (down)	Cell Signaling
JNK1/2	54 kDa (up) 46 kDa (down)	Cell Signaling
P-P38	43 kDa	Cell Signaling
P38	40 kDa	Cell Signaling
